# College and Hospital Notes

**Published:** 1908-05

**Authors:** 


### COLLEGE AND HOSPITAL NOTES.

---

THE University of Buffalo will hold its sixty-third annual commencement exercises Friday, May 29, 1908. Commencement week will be inaugurated on Tuesday evening, May 26, with a reception by the council of the University and the governing faculty of the medical department at which short addresses will be given. The classes of '48, '53, '58, '78, '83, '88 and '98 will hold reunions. A smoker will be given after the regular exercises at which refreshments will be served.

Wednesday and Thursday mornings and afternoons will be devoted to clinics at the Buffalo General Hospital. Luncheons, smokers, receptions, theater parties and other entertainments will fill the vacant hours.

A museum of anatomic, physiologic and pathologic specimens will be on exhibition during the entire week. Members of the alumni association are requested to make contributions to the exhibit. The annual dues of $1.00 should be paid to the treasurer, Herman K. DeGroat, either by mail or in person.

---

The alumni of the medical department of Tulane University of Louisiana will celebrate the Stanford E. Chaille golden jubilee at New Orleans, May 19, and 20, 1908, during which it is proposed to raise a memorial fund to establish a chair of physiology or a chair of hygiene to be named after Dr. Chaille.

Dr. L. S. McMurtry, of Louisville, Ky., (Tulane, '73) will deliver the principal address on behalf of the alumni May 19. This meeting to celebrate the fiftieth year of teaching service of Professor Chaille will be a memorable one in the history of medical colleges in this country. Every alumnus is invited to attend and to contribute to the fund.

---

The Lockport City Hospital was formally turned over to the municipal authorities Saturday, April 18, 1908. The formal opening of the institution took place April 21.

The hospital is the only one in the state which is directly under municipal control, and it will be in the nature of an experiment for the first few years as to whether a municipality can run a hospital as well as a hospital association.

The building is on East avenue, near the city line, and of the first $15,000 spent on the structure, the city gave $5,000; the women of the Hospital Aid Association raised $5,000, and the late Augustus Keep gave $5,000.

---

The German Deaconess's Hospital on Kingsley street, Buffalo, reopened April 4, 1908, after being closed several weeks for improvements. A training school for nurses has been established under the auspices of the Fliedner League. Sister Superior Anna Dalchow will be in charge of the deaconesses. Miss Gertrude Breslin will have charge of the nurses and Miss Frances Welker, formerly of the Titusville, Pa., Hospital, will be the assistant superintendent. Miss Breslin has held like positions in New York, Louisville, Nashville and Detroit and comes highly recommended to the directors of the Deaconess's Hospital. A new ambulance service has been provided and the hospital will take ambulance calls day and night. The left wing of the building will continue as a home for the aged.

---

The Marine Hospital project is assuming shape. The government has asked for bids from contractors, which are returnable May 21, 1908. The specifications demand that the contractor to whom the contract is awarded shall insure its completion by March 31, 1910.

---

Notice to Alumni of the Tulane Medical Department.—It is important that all graduates of Tulane intending to be present at the meeting of the A. M. A. in Chicago, June 2 to 5, should write at once to Dr. Hugh B. Williams, No. 100 State St., for information concerning the gathering of the Alumni on June 2. Tulane headquarters will be at the Auditorium Hotel and Almuni are urged to call upon their arrival for information. This is important.

